# Macrophage-dependent tumor cell transendothelial migration is mediated by Notch1/Mena^INV^-initiated invadopodium formation

**DOI:** 10.1038/srep37874

**Published:** 2016-11-30

**Authors:** Jeanine Pignatelli, Jose Javier Bravo-Cordero, Minna Roh-Johnson, Saumil J. Gandhi, Yarong Wang, Xiaoming Chen, Robert J. Eddy, Alice Xue, Robert H. Singer, Louis Hodgson, Maja H. Oktay, John S. Condeelis

**Affiliations:** 1 Department of Anatomy and Structural Biology Albert Einstein College of Medicine of Yeshiva University, Bronx, NY 10461, United States; 2 Gruss Lipper Biophotonics Center Albert Einstein College of Medicine of Yeshiva University, Bronx, NY 10461, United States; 3 Department of Pathology Albert Einstein College of Medicine of Yeshiva University, Bronx, NY 10461, United States.

## Abstract

The process of intravasation involving transendothelial migration is a key step in metastatic spread. How the triple cell complex composed of a macrophage, Mena over-expressing tumor cell and endothelial cell, called the tumor microenvironment of metastasis (TMEM), facilitates tumor cell transendothelial migration is not completely understood. Previous work has shown that the physical contact between a macrophage and tumor cell results in the formation of invadopodia, actin-rich matrix degrading protrusions, important for tumor cell invasion and transendothelial migration and tumor cell dissemination. Herein, we show that the macrophage-induced invadopodium is formed through a Notch1/Mena^INV^ signaling pathway in the tumor cell upon macrophage contact. This heterotypic tumor cell – macrophage interaction results in the upregulation of Mena^INV^ through the activation of MENA transcription. Notch1 and Mena^INV^ expression are required for tumor cell transendothelial migration, a necessary step during intravasation. Inhibition of the Notch signaling pathway blocked macrophage-induced invadopodium formation *in vitro* and the dissemination of tumor cells from the primary tumor *in vivo*. Our findings indicate a novel role for Notch1 signaling in the regulation of Mena^INV^ expression and transendothelial migration and provide mechanistic information essential to the use of therapeutic inhibitors of metastasis.

Metastasis is the primary cause of morbidity and mortality of breast cancer patients. The process of breast cancer metastasis requires tumor cells to migrate towards blood vessels where they intravasate. Intravasation of tumor cells during metastasis remains poorly understood, although it has become increasingly apparent that components of the tumor microenvironment such as macrophages, contribute to the efficacy of this process. Within invasive breast carcinoma we have identified multicellular microanatomical structures called the tumor microenvironment of metastasis (TMEM) that serve as the functional sites of tumor cell intravasation, and have been validated as a prognostic marker of metastatic outcome in patients^1^,^2^,^3^,^4^,^5^. Each TMEM site is comprised of a Mena over-expressing tumor cell, a peri-vascular macrophage and an endothelial cell of a blood vessel in direct contact with each other. It is at these sites that transient vascular- permeability events and intravasation of breast tumor cells uniquely occur in breast tumors^2^. The importance of the direct contact of these three cell types and signaling between these cells during the function of TMEM in intravasation has not yet been explored.

Migratory and disseminating breast tumor cells at TMEM over-express the ENA/Vasp family member Mena (mammalian-ENA) when compared to normal breast epithelium^4^,^6^. Overexpression of Mena, an actin regulatory protein, is also seen in other cancers of epithelial origin including pancreatic, lung, cervical and colon cancers^7^. Mena is alternatively spliced during tumor progression resulting in the expression of multiple isoforms encoded by the same gene^7^. The expression of the different isoforms is associated with various cell phenotypes. Predominate expression of the Mena11a isoform confers an epithelial, cohesive, non-invasive cell behavior; while the over-expression of Mena^INV^ results in a highly motile, invasive phenotype^8^,^9^,^10^.

The number of TMEM sites in patient samples positively correlates with the abundance of Mena^INV^ mRNA, whereas Mena11a demonstrates a negative correlation in the same patient cohort^1^. Previous work has demonstrated that the expression of the Mena11a isoform is regulated by Twist signaling^11^, but despite the numerous studies indicating the over-expression of Mena^INV^ in invasive-intravasation-competent tumor cells *in vivo*, the signals regulating the expression of the Mena^INV^ isoform have remained unknown.

In addition to the macrophage-regulated vascular permeability events that contribute to tumor cell intravasation and metastasis, tumor cells form an invasive actin-rich invadopodium that enables the tumor cell to degrade extra-cellular matrix proteins and migrate, invading across barriers like blood vessel walls and intravasate^12^,^13^,^14^. Invadopodia are required for tumor cell transendothelial migration, a necessary step for intravasation^13^. Although it has been generally appreciated that invadopodia are important for tumor cell migration and intravasation it is not completely understood how^15^,^16^. There is evidence in multiple *in vivo* models such as chicken chorioallantoic membrane (CAM) models, tumor xenographs in mice and zebrafish and transgenic mouse models that invadopodia can be observed in mammalian cells *in vivo* and that these structures play a critical role in the cells ability to breech the basement membrane for invasion^17^,^18^,^19^,^20^. In addition, the depletion of critical invadopodial components *in vivo* reduces the numbers of circulating tumor cells and metastasis^12^. Interestingly, we have previously shown that the direct interaction of a tumor cell and macrophage results in the formation of the tumor cell invadopodium that is required for transendothelial migration of tumor cells and this cannot be mimicked with macrophage-conditioned medium^13^. Therefore, a direct contact event between tumor cells and macrophages results in a signal inducing the formation of invadopodia.

A major signaling pathway that is involved in cell contact-mediated communication is the Notch signaling pathway. In addition to critical roles in development, Notch signaling has been implicated in cancers such as breast, lung and pancreatic cancers and leukemia, where activation of Notch pathways can promote proliferation, prevent differentiation, and promote metastasis^21^,^22^,^23^,^24^. Disruption of the Notch signaling pathways can affect cell growth, cell fate, angiogenesis and apoptosis. In tumor cells, activation of Notch upon homotypic cell contact triggers invadopodium formation under hypoxia conditions^25^.

Herein, we explore the contribution of the Notch signaling pathway to TMEM function; in particular macrophage-dependent tumor cell invadopodium formation and its relationship to Mena expression during transendothelial migration and tumor cell dissemination.

## Results

### Notch1 signaling is required for macrophage-induced formation of invadopodia in tumor cells

**Figure 1:**
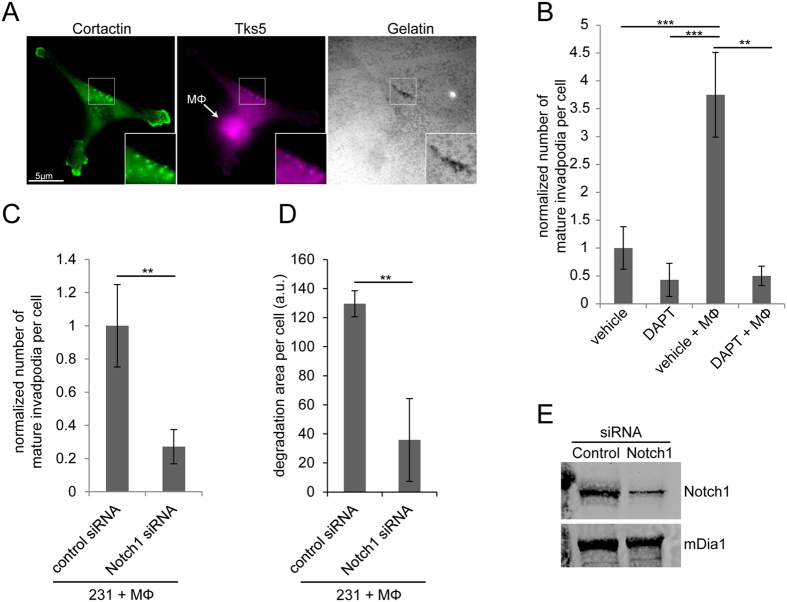
Macrophage – induced tumor cell invadopodia require Notch1 signaling. (**A**) Immunofluorescence of MDA-MB-231 tumor cell in contact with BAC1.2F5 macrophage plated on 405-gelatin. Tumor cells were stained for Tks5 (purple) and cortactin (green) to identify invadopodium cores (insert is zoom of mature invadopodia). Position of the macrophage is indicated by the arrow. (**B**) Quantitation of the number of mature invadopodia per cell in MDA-MB-231 tumor cells plated alone or with BAC1.2F5 macrophages. Cells were treated with vehicle or DAPT γ- secretase inhibitor. (**C**) Quantitation of the number of mature invadopodia per cell in MDA-MB-231 tumor cells treated with control or Notch1 siRNA plated with BAC1.2F5 macrophages. (**D**) Quantitation of the area of matrix degradation in control and Notch1 siRNA treated MDA-MB-231 cells plated with BAC1.2F5 macrophages. (**E**) Western blot of MDA-MB-231 cells treated with control and Notch1 siRNA demonstrating knockdown efficiency. mDia1 (mammalian homolog of Drosophila diaphanous) was used as a loading control. *P < 0.05, **P < 0.005, ***P < 0.0005.

To evaluate if Notch signaling is required for macrophage – induced invadopodium formation (Fig. 1A), cells were treated with DAPT, a γ-secretase inhibitor, which inhibits intracellular Notch signaling by preventing its cleavage into the active NICD^26^. A mature invadopodium is defined herein as having cortactin and Tks5 positive staining as well as being co-localized with a discreet area of matrix degradation. Tks5 is required for anchoring the invadopodium core to the plasma membrane via its binding to PI (3, 4) P2^27^ and its association with these other two markers is a definitive identifier of mature invadopodia. In the absence of macrophages and in serum-starved conditions, DAPT treatment has no significant effect on invadopodium assembly by MDA-MB-231 human breast tumor cells (Fig. 1B). When BAC1.2F5 macrophages are added to the MDA-MB-231 culture there is a significant increase in the number of mature invadopodia per tumor cell but the addition of DAPT to these co-cultures prevents the macrophage-mediated induction of invadopodia (Fig. 1B).

Increased Notch1 signaling is associated with a greater chance of metastasis and poor prognosis^23^. Therefore, to determine if Notch1 receptor is important in macrophage–induced invadopodium formation we used siRNA mediated knockdown of Notch1. Knockdown of Notch1 receptor in MDA-MB-231 cells resulted in a significant reduction in macrophage-induced invadopodia as well as a significant reduction in invadopodium-associated matrix degradation to baseline levels seen in the absence of macrophages (Fig. 1C–E). Notch1 inhibition had no significant effect on steady sate invadopodium formation in the absence of macrophages (Supplemental Figure 1A and B). Thus, we conclude that Notch1 is required for macrophage-induced invadopodium assembly and function. These data indicate a novel signaling pathway involving heterotypic cell-cell communication of Notch1 in macrophage-induced invadopodium formation in tumor cells.

### Notch1 signaling is required for macrophage – tumor cell contact-induced Mena^INV^ expression

**Figure 2:**
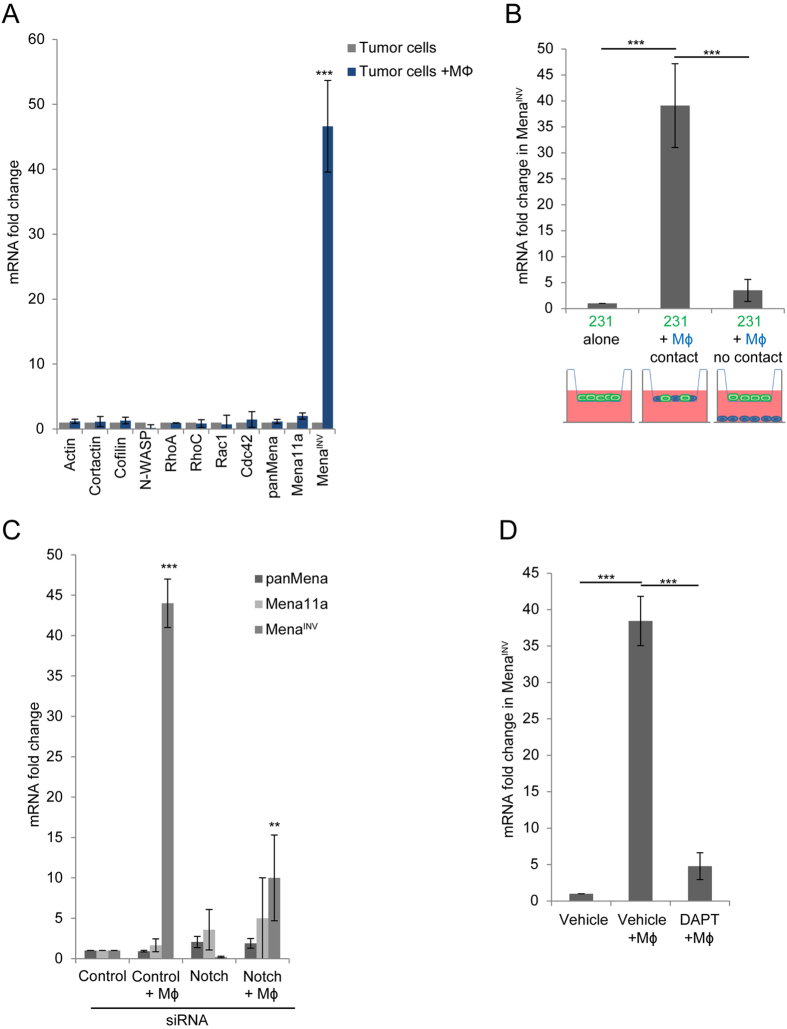
Macrophage – tumor cell contact results in a Notch1 dependent upregulation in Mena^INV^ mRNA expression in tumor cells. (**A**) qRT-PCR of invadopodia pathway components in MDA-MB-231 cells plated alone or with BAC1.2F5 macrophages (Mϕ). (**B**) qRT-PCR of Mena^INV^ in MDA-MB-231 cells plated in transwells alone, co-cultured in top well of transwells in contact with BAC1.2F5 macrophages (Mϕ) or plated on opposite side of transwells (no contact). (**C**) qRT-PCR of Mena^INV^ in MDA-MB-231 cells treated with control or Notch1 siRNA plated alone or with BAC1.2F5 macrophages. (**D**) qRT-PCR of Mena^INV^ in MDA-MB-231 cells treated with vehicle or DAPT γ- secretase inhibitor plated alone or with BAC1.2F5 macrophages. *P < 0.05, **P < 0.005, ***P < 0.0005.

Notch1 has well defined roles in the regulation of gene and protein expression at the transcriptional level, although a role for Notch1 in the regulation of the macrophage-induced invadopodium pathway is unknown. We sought to determine if macrophage – induced Notch1 signaling in tumor cells results in expression changes in genes with known roles in invadopodium formation and function. We performed a qRT-PCR analysis to determine changes in mRNA levels of the known invadopodium pathway regulatory proteins (Fig. 2A). The co-culture of MDA-MB-231 tumor cells with macrophages resulted in no significant mRNA changes in actin, cortactin, cofilin, N-WASp or the RhoGTPases Rac1, Cdc42, RhoA and RhoC, which all have well defined roles in invadopodia regulation^12^,^28^,^29^,^30^,^31^,^32^,^33^. There was no significant change in the level of total (pan) Mena mRNA levels which are very abundant compared to Mena^INV^ (Supplemental Figure 2B), but the Mena^INV^ isoform was highly upregulated (~45x) while Mena11a isoform levels remain unchanged. This finding is of great interest because high expression of the Mena^INV^ isoform has been implicated in the increased motility, invadopodium assembly and invasion by tumor cells^1^,^8^,^34^,^35^.

There is a well described paracrine signaling loop between human breast or mouse mammary tumor cells and macrophages^1^,^35^,^36^. Due to this well-defined soluble signaling pathway between tumor cells and macrophages, we wanted to confirm that the increase in Mena^INV^ upon co-culture with macrophages was a result of cell-cell contact and not soluble signaling factors. We performed co-culture experiments utilizing 3 μm pore transwells, which allow for the passage of soluble factors but not the tumor cells. Tumor cells were either plated in the transwell alone, with the two cell types mixed in the top of the well, or with macrophages added to the bottom of the well to prevent tumor cell – macrophage contact (Fig. 2B). When the cell types were mixed together in the top of the transwell, allowing cell-cell contact, we observed the increase in Mena^INV^ mRNA whereas when the cells were plated on opposite sides of the transwell, allowing for the exchange of soluble factors but not direct contact, no significant change in Mena^INV^ mRNA was observed.

To confirm that the increase in Mena^INV^ mRNA is regulated by the macrophage-tumor cell contact initiated Notch1 signaling, we treated the tumor cells with Notch1 siRNA or DAPT. We found that both had the ability to abrogate the increase in Mena^INV^ mRNA during co-culture of the two cell types (Fig. 2C and D).

To determine if these events are macrophage-contact specific, we co-cultured tumor cells at high density to induce tumor cell-tumor cell contact, tumor cell and macrophage contact or tumor cells and endothelial cell contact. These are the three different cell types that comprise TMEM sites. The only contact that was able to induce an increase in Mena^INV^ expression was the tumor cell and macrophage co-culture (Supplemental Figure 2A). In addition, the contact between tumor cells and HUVEC endothelial cells did not result in an increase in mature invadopodia or matrix degradation (Supplemental Figure 2B and C).

**Figure 3:**
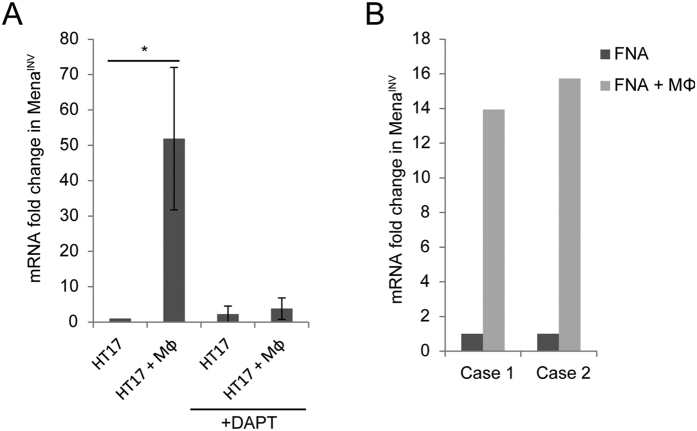
Macrophage contact induces a Notch dependent upregulation of Mena^INV^ in primary human breast tumor cells. (**A**) qRT-PCR of Mena^INV^ of primary FNA tumor cells from HT17 triple negative human tissue transplants in SCID mice cultured alone or with BAC1.2F5 macrophages (n = 3 mice) in the presence of absence of DAPT. (**B**) qRT-PCR of Mena^INV^ from primary FNA tumor cells from two independent patients cultured alone or with BAC1.2F5 macrophages. Case 1 was an ER+ tumor and case 2 was an ER − tumor.

In addition to the MDA-MB-231 cells, we confirmed the macrophage-induced expression of Mena^INV^ in primary tumor cells. Tumor cells were obtained by fine needle aspiration biopsy (FNA), which yields ~97% pure tumor cell samples^1^. Cells obtained by FNA from triple negative human tumor tissue transplants (HT17)^1^,^37^ were plated with or without macrophages for 6 hours. Although a subset of tumor cells *in vivo* express elevated Mena^INV^, when cells were cultured with macrophages there is a significant increase in Mena^INV^ mRNA (Fig. 3A). When cells are plated with macrophages in the presence of DAPT there was an inhibition of Mena^INV^ expression (Fig. 3A), indicating a requirment for Notch signaling in primary tumor cells.

Primary cancer cells were also obtained by FNA from two human invasive ductal carcinomas immediately after surgical resection (Fig. 3B, patient #1 had ER+ disease and patient #2 had ER- disease). In cells from these 2 patient samples we also observed a dramatic increase in Mena^INV^ mRNA when co-cultured with macrophages (Fig. 3B). These data indicate a novel pathway for the upregulation of the pro-migratory/invasive Mena^INV^ isoform of Mena that is common to breast cancer cells with different ER expression status.

### Macrophage – tumor cell contact turns on Notch-dependent MENA gene transcription and Mena^INV^ protein expression

The above results indicate that a Mena isoform switch is taking place upon macrophage contact because the panMena and Mena11a mRNA levels remained unchanged while Mena^INV^ levels increase dramatically. This expression switch of isoforms presumably requires the transcription of the MENA gene, where the newly transcribed RNA would be spliced to include the Mena^INV^ exon.

**Figure 4:**
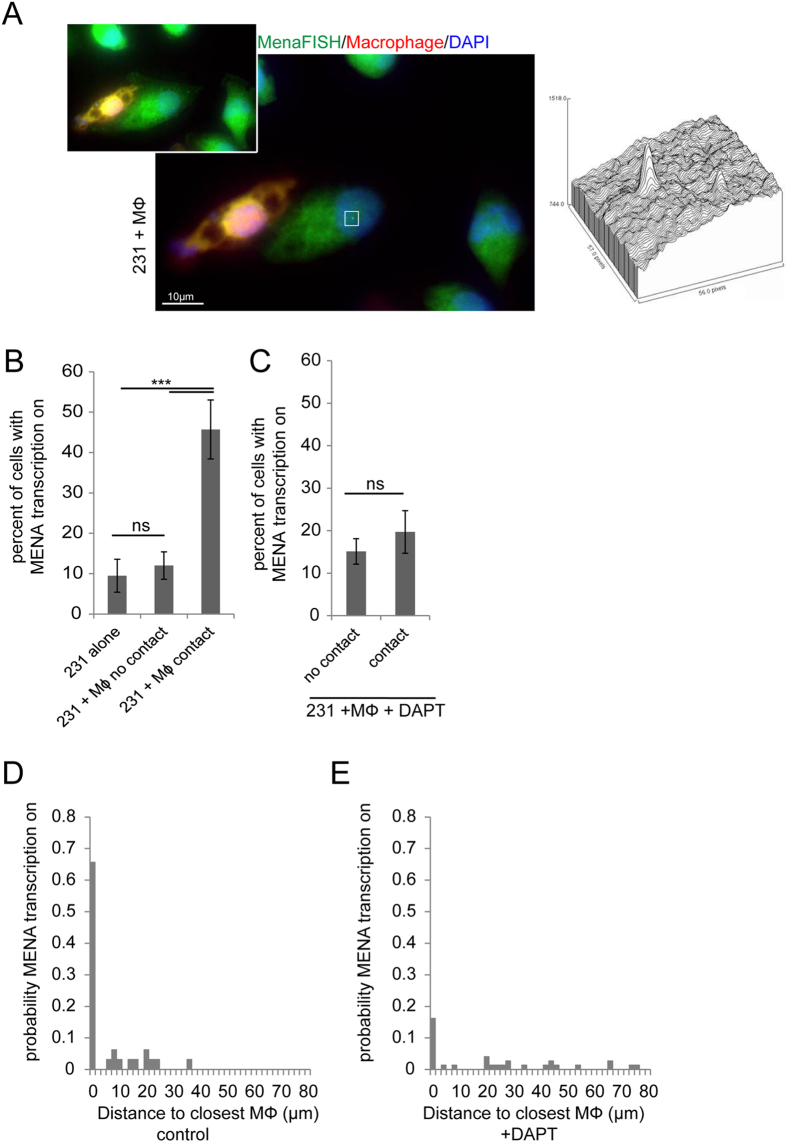
Macrophage- tumor cell contact increases MENA transcription. (**A**) MDA-MB-231 cells plated with BAC1.2F5 macrophages for 60 min. Macrophages are labeled with cell tracker red. Cells were labeled with FISH probes that recognize all Mena isoforms (panMena) and DAPI. Inset shows z-section of direct contact of tumor cell with macrophage. White box is around the dot which is an active MENA transcription site. Histogram of FISH fluorescence intensity within white box showing signal to noise intensity of transcription site. (**B**) Quantitation of the percent of tumor cells with active MENA transcription when MDA-MB-231 cells are plated alone, with BAC1.2F5 macrophages (Mϕ) not in contact and with Mϕ in contact with tumor cells. (**C**) Quantitation of the percent of tumor cells with active MENA transcription when MDA-MB-231 cells with BAC1.2F5 macrophages (Mϕ) not in contact and with Mϕ in contact with tumor cells in the presence of DAPT γ- secretase inhibitor. (**D**) The distance of tumor cells from macrophages in the population of cells with MENA transcription sites being expressed (probability). (**E**) The distance of tumor cells from macrophages in the population of cells with MENA transcription sites being expressed in the presence of DAPT (normalized for the number of cells with MENA being expressed). *P < 0.05, **P < 0.005, ***P < 0.0005, *ns* = non-significant.

To test the hypothesis that the increase in Mena^INV^ mRNA seen in tumor cells in contact with macrophages is associated with increased Mena gene transcription, we designed fluorescent *in-situ* hybridization (FISH) probes to MENA. The different Mena isoforms are splice variants transcribed by the same gene^6^,^11^; therefore the probes designed recognize the full length MENA gene transcript (Supplemental Figure 3). Macrophages were labeled with cell tracker red and co-cultured with tumor cells for 60 minutes (Fig. 4A). When MDA-MB-231 tumor cells are cultured alone, only ~10% have an active MENA transcription site, as seen by FISH (Fig. 4B). When tumor cells and macrophages are plated in co-culture for 1 hour, tumor cells that are not in contact with macrophages still only display ~10% of cells actively transcribing MENA, while tumor cells touching a macrophage have over 45% of cells with active transcription sites (Fig. 4A and B). Figure 4A shows a tumor cell in direct contact with a macrophage displaying active MENA transcription, and a tumor cell not in contact with a macrophage displaying no active MENA transcription site. The addition of DAPT to the tumor cell-macrophage co-culture prevented the macrophage induction of MENA transcription, even when the tumor cells were in direct contact with macrophages (Fig. 4C).

In addition, we measured the probability of active MENA transcription as a function of the distance of a tumor cell to the nearest macrophage in control conditions or in the presence of DAPT (Fig. 4D and E). When tumor cells are touching macrophages (0 μm distance), the probability of active MENA transcription is ~0.65, a high probability of transcription. As the distance becomes greater than 35 μm away, there is zero probability that the tumor cell has an active MENA transcription site. In the presence of DAPT treatment, tumor cells show a scattered pattern of low level transcription versus distance from a macrophage indicating that by blocking Notch signaling we have inhibited the macrophage contact induced MENA transcription.

These FISH data and the qPCR data above indicate that when tumor cells come into direct contact with a macrophage there is a rapid Notch-dependent induction of MENA transcription.

**Figure 5:**
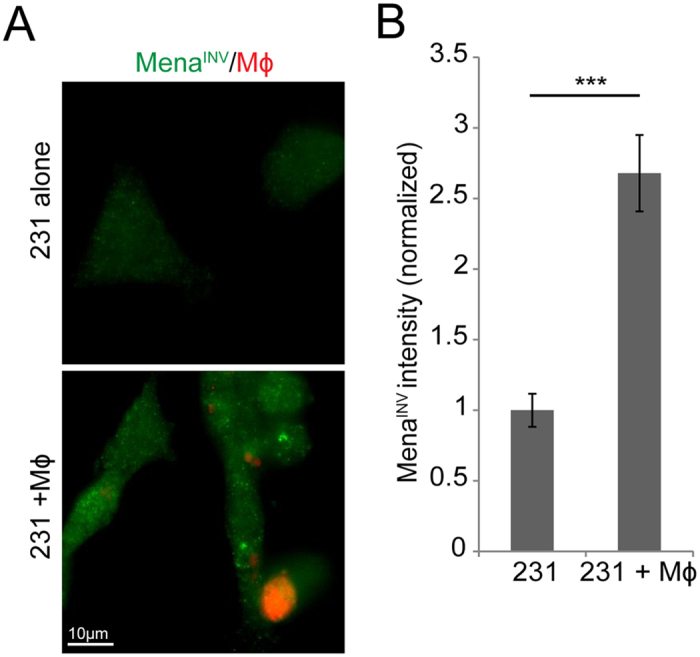
Macrophage-tumor cell contact increases Mena^INV^ protein expression in tumor cells. (**A**) Immunofluorescence of MDA-MB-231 cells plated alone or with BAC1.2F5 macrophages (cell tracker red) stained with a Mena^INV^ specific antibody. (**B**) Quantitation of the average pixel intensity of Mena^INV^ staining in MDA-MB-231 cells plated alone or with BAC1.2F5 macrophages.

To determine if the macrophage activation of MENA transcription and the increased accumulation of Mena^INV^ mRNA results in increased Mena^INV^ protein in the tumor cells, we developed a polyclonal anti- Mena^INV^ specific antibody (Supplemental Figure 4). MDA-MB-231 cells plated alone or in co-culture with macrophages for 16 hours were stained using the newly developed Mena^INV^-specific antibody (Fig. 5A). The average intensity was measured with Mena^INV^ staining in tumor cells alone or those cultured with macrophages. When cultured with macrophages, tumor cells show a significant increase in Mena^INV^ protein staining intensity (Fig. 5). This data indicates that the Notch-mediated increase in Mena^INV^ mRNA observed in tumor cells in contact with macrophages is translated into an increase in Mena^INV^ protein levels in the tumor cells.

### Mena^INV^ and Notch1 are required for invadopodium-driven transendothelial migration

The above results demonstrate that macrophage – induced tumor cell invadopodium formation requires Notch1 signaling. We also know from previous work that macrophages induce transendothelial migration of tumor cells both *in vitro*^13^ and *in vivo*^2^, and that Mena^INV^ is required for transendothelial migration^1^. Therefore, we hypothesized that Notch1 and Mena^INV^ are both required for the formation of the invadopodia necessary for tumor cells to undergo transendothelial migration.

**Figure 6:**
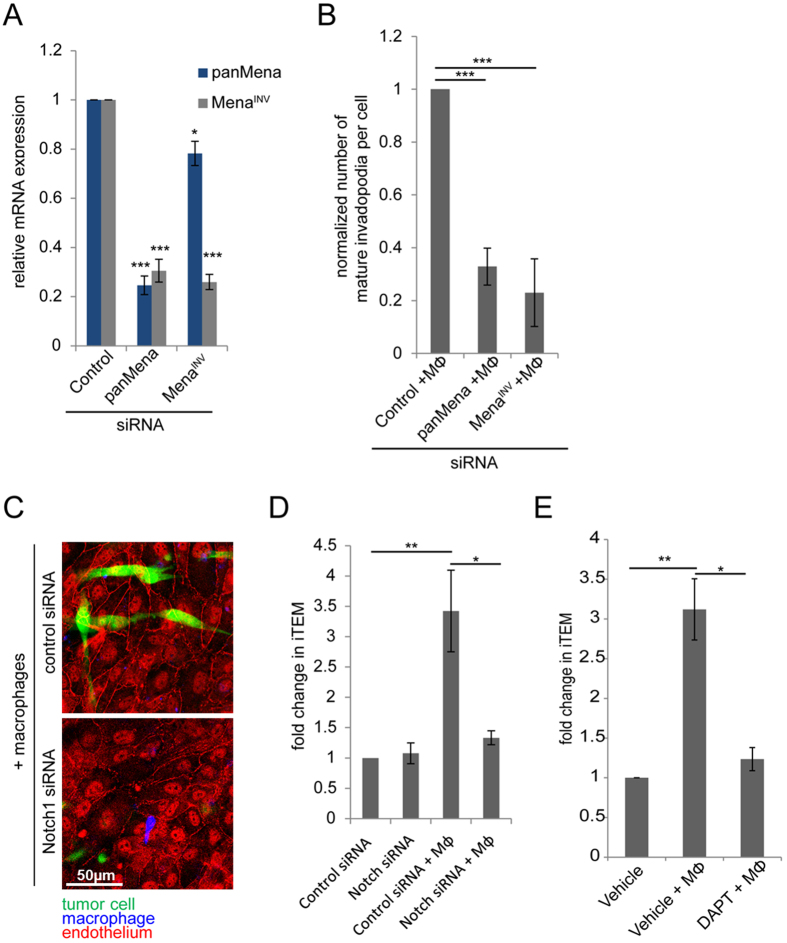
Mena^INV^ and Notch1 are required for macrophage – induced invadopodium assembly and transendothelial migration. (**A**) Quantitation of the relative mRNA expression in MDA-MB-231 cells treated with control, panMena or MenaI^NV^ siRNA in the absence of macrophages. (**B**) Quantitation of the relative number of invadopodia in MDA-MB-231 cells treated with control, panMena or Mena^INV^ siRNA in the presence of macrophages. (**C**) Representative apical z-section of intravasation-directed transendothelial migration (iTEM) assay of tumor cells (green) and macrophages (blue, white arrows). Tumor cells were treated with control or Notch1 siRNA. Endothelial HUVEC cells were stained for ZO-1 (red). Shown is an “en face” view of the apicial side of the transwell, therefore the green-labeled tumor cells have crossed the endothelial monolayer and are on the ‘bottom’ or apical side of the transwell positioning them above the endothelial cells from this vantage point. (**D**) Quantitation of intravasation-directed is defined as subluminal to luminal transendothelial migration (iTEM) activity of MDA-MB-231 cells plated on the endothelium either alone or with BAC1.2F5 macrophages. Tumor cells were treated with control or Notch1 siRNA. (**E**) Quantitation of iTEM activity of MDA-MB-231 cells plated alone or with BAC1.2F5 macrophages with vehicle or DAPT treatment. *P < 0.05, **P < 0.005, ***P < 0.0005.

Specific knockdown of Mena^INV^ significantly reduces the number of mature invadopodia formed upon macrophage-tumor cell contact (Fig. 6A and B). Knockdown of panMena results in the knockdown off all Mena isoforms, therefore also depleting Mena^INV^. Interestingly, depletion of total Mena has no additive effect over the Mena^INV^ knockdown (Fig. 6A and B). Therefore, Mena^INV^ is the key isoform that regulates macrophage-induced invadopodia.

To determine the role of Notch1 in tumor cell intravasation-directed transendothelial migration (iTEM) activity, we either depleted Notch1 in tumor cells with siRNA or inhibited Notch signaling with DAPT and quantified the tumor cell iTEM activity in the presence and absence of macrophages. Notch1 depletion had no effect on the iTEM activity of tumor cells alone, but either depletion of Notch1 with siRNA or its inhibition with DAPT inhibited macrophage-induced tumor cell iTEM activity (Fig. 6C–E).

### Notch1 inhibition decreases macrophage-dependent invadopodium formation *in vitro* and the dissemination of photo-converted tumor cells *in vivo*

**Figure 7:**
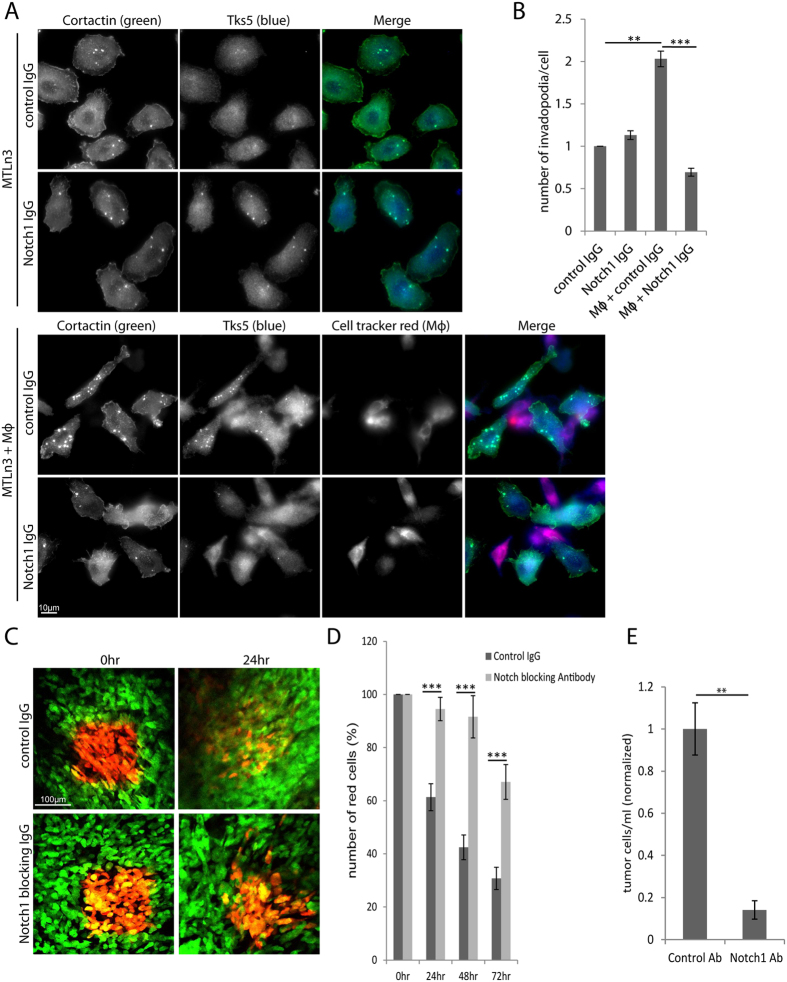
Notch1 inhibition decreases macrophage-dependent invadopodium formation *in vitro* and the dissemination of tumor cells *in vivo*. (**A**) Immunofluorescence images of MTLn3 tumor cells plated in the presence or absence of BAC1.2F5 macrophages (Mϕ) and treated with control IgG or Notch1 blocking IgG. Macrophages were labeled with cell tracker red and cells were stained for cortactin and Tks5. (**B**) Quantitation of the relative number of invadopodia per cell in MTLn3 cells plated alone or with Mϕ and treated with control IgG or Notch1 blocking IgG. (**C**) Dendra2-MTLn3 xenograph mammary tumors in SCID nice were imaged using a mammary imaging window. Regions in vascularized areas were photo-converted in mice treated with control IgG or Notch1 blocking IgG. Photo-converted cells were tracked at 0 and 24 hours. (**D**) Quantitation of the percent of red photo-converted cells remaining in the photo-converted region at 24 hours measures amount of intravasation. (**E**) Quantitation of the circulating tumor cells from SCID mice with MTLn3 xenographs. Mice were treated with control IgG or Notch1 blocking IgG. *P < 0.05, **P < 0.005, ***P < 0.0005.

Formation of invadopodia *in vitro* as well as invasion *in vivo* has been well characterized using the rat mammary adencocarcoma MTLn3 cells. Like MDA-MB-231 cells, MTLn3 cells can form invadopodia and undergo migration *in vivo* when they upregulate Mena^INV^ expression^6^,^35^. Here, we tested their ability to form macrophage-induced invadopodia. When MTLn3 cells are co-cultured with macrophages, we observed a highly significant increase in formation of invadopodia (Fig. 7A and B). As an additional method of inhibiting Notch1 signaling applicable to the MTLn3 cells, we treated MTLn3 cells with a Notch1 specific blocking antibody or control isotype IgG. Cells co-cultured with macrophages and treated with the Notch1 blocking antibody demonstrate significantly fewer invadopodia (Fig. 7A and B). These data demonstrate that the macrophage-induced invadopodia can be stimulated in a different well-characterized invasive and metastatic tumor cell line and this is Notch1 dependent.

We utilized MTLn3 cells expressing photo-convertible Dendra2 to measure the effects of Notch1 inhibition on loss of tumor cells from the primary tumor *in vivo* as described previously^12^,^38^. Peri-vascular regions within the MTLn3 – Dendra2 tumors were photo-converted and were tracked every 24 hours over a 72 hour period to measure intravasation as described previously^10^,^14^,^38^ (Fig. 7 C and D). Mice were treated daily with 1 mg/kg control IgG or Notch1 blocking IgG. In control treated mice, there was a significant decrease every 24 hour period in the percent of photo-converted-Dendra2 cells remaining in the region^10^,^14^,^38^. In mice treated with the Notch1 blocking antibody there was a significant reduction in the percent of cells that were leaving the photo-converted regions of the tumor, indicating these tumor cells were less efficient at leaving the primary tumor.

To find out if cells leaving the tumor site were correlated with circulating tumor cells, mice with MTLn3 xenographs were treated with control or Notch1 blocking IgG for 6 hours and the circulating tumor cells were measured after treatment. There was a significant decrease in the number of circulating tumor cells in mice that were treated with the Notch1 blocking antibody compared to control treated mice (Fig. 7E), consistent with the idea that transendothelial migration is involved in CTC (Circulating Tumor Cells) number. These data indicate a role for Notch1 signaling in tumor cells leaving the primary tumor.

## Discussion

Tumor cell dissemination via blood vessels in breast tumors requires tumor cells to undergo transendothelial migration. This occurs uniquely at TMEM sites where macrophages are in direct contact with tumor cells and endothelial cells^2^. In this study we define the molecular mechanism by which the direct contact between macrophages and tumor cells leads to invadopodium formation and transendothelial migration. We show that these events require the Notch1 receptor on the tumor cell and that Notch1 signaling induces Mena^INV^ expression via activation of transcription. We found that Notch1 is required for transendothelial migration of tumor cells and dissemination of tumor cells from the primary tumor. These results are consistent with previous work showing that Mena^INV^ expression is required for transendothelial migration by tumor cells^1^, that the knockdown of Mena^INV^ inhibits macrophage-mediated transendothelial migration of tumor cells^1^ and, conversely, that Mena^INV^ over-expression drives invadopodium assembly and function, and transendothelial migration^10^.

The relative expression of Mena^INV^ to that of Mena11a is associated with TMEM assembly, metastatic recurrence and death of breast cancer patients^1^,^39^,^40^. This is significant because TMEM is the doorway for intravasation of tumor cells into the blood vessels in breast tumors and the number of TMEM sites in a breast tumor is highly predictive of risk of distant recurrence in patients^3^,^4^. Previous work demonstrated the signals that lead to decreased expression of Mena11a^11^. However, until now the signals responsible for the induction of Mena^INV^ were not known. Here we show that the expression of the Mena^INV^ isoform is induced by activation of Notch1 signaling in tumor cells. In this regard our findings indicate a novel role for Notch1 signaling in the regulation of Mena^INV^ expression and transendothelial migration at TMEM sites.

The induction of invadopodia in tumor cells by macrophages identifies an important step in tumor cell dissemination. Invadopodia are F-actin-rich protrusions on tumor cells capable of degrading extracellular matrix and assisting in tumor cell chemotaxis and migration^41^. Invadopodium initiation has been described during tumor hypoxia emphasizing the importance of the tumor microenvironment in the regulation of invasive protrusion initiation and function^25^. While tumor cells form invadopodia in response to both growth factor and integrin signaling^29^,^42^,^43^ our studies described herein show, for the first time, that macrophages can initiate invadopodium assembly and this requires Notch signaling uniquely. These results emphasize selectivity differences in how invadopodia are initiated, and the importance of the tumor microenvironment in determining the different invadopodium functions that result from differences in how invadopodia are initiated; chemotaxis during invasive migration involving invadopodium initiation in response to growth factors^44^, fibronectin directed invasion involving invadopodium initiation by integrin beta-1^43^, and transendothelial migration as described here involving invadopodium initiation via Notch signaling in response to heterotypic cancer cell-macrophage contact.

The analysis of the gene expression pattern of migratory and disseminating tumor cells in breast tumors revealed an “invasion signature” that is associated with distant recurrence in breast cancer patients^5^,^35^,^37^. A prominent pathway in the invasion signature is the Mena–Cofilin pathway that regulates actin polymerization during chemotaxis and invasion of tumor cells^9^,^34^,^45^ and which is associated with poor outcome in breast cancer patients^39^,^40^,^46^. Investigation of the gene expression pathways of the invasion signature revealed that differentially spliced Mena^INV^ is upregulated, while the invasion-suppressing Mena11a isoform is downregulated in migratory/disseminating tumor cells^6^,^8^,^9^,^10^,^35^,^37^,^47^. This isoform splicing pattern of Mena (Mena^INV-high^/Mena11a^low^) is associated with directional cell migration towards chemotactic factors such as EGF and HGF, matrix degradation, TMEM assembly and transendothelial migration^8^,^10^,^34^,^35^,^37^,^48^, as well as poor outcome in breast cancer patients^5^,^39^,^40^. In addition, Mena^INV^ dramatically increases the sensitivity of receptor tyrosine kinases to their ligands EGF, IGF1 and HGF to increase cell protrusion and locomotion of tumor cells toward blood vessels^9^,^10^,^49^.

Previous studies have shown that heterotypic interactions among cells surrounding intratumoral vasculature can promote cancer cell dissemination. For example, fibroblast-derived lysly oxidase, a matrix cross-linking enzyme that stiffens collage fibers, driven by myeloid cell-derived TGFβ, promotes cancer metastasis^50^. Another study demonstrated involvement of endosialin-expressing pericytes in cancer cell transendothelial migration and dissemination^51^. Endothelial cells are also actively involved in regulation of cancer cell dissemination. The interaction of CXCR12 (SDF-1), secreted by endothelial cells, with tumor cell expressed CXCR4 is sufficient to stimulate transendothelial migration of the tumor cells^52^. Interestingly, the induction of SDF-1 in endothelial cells seems to be mediated by hypoxia. CXCL12 (SDF-1) can also be expressed by tumor cells and it results in increased macrophage and microvessel density and *in vivo* invasiveness^53^. Increased macrophage density has been shown to contribute to cancer cell invasiveness and metastasis as shown by several laboratories including ours^1^,^2^,^13^,^54^. The presence of macrophages greatly enhances the ability of both tumor cell lines and primary breast tumor cells to undergo intravasation-directed transendothelial migration (iTEM) and that iTEM requires Mena^INV^ expression and invadopodium formation^1^,^10^,^13^. Our study here has added the mechanistic insight into how Mena^INV^ upregulation is achieved in tumor cells and opens the future exploration of how different macrophage ligands might activate Notch signaling on tumor cells to lead to phenotypes associated with tumor metastasis.

Our results are directly relevant to how Mena isoform expression and TMEM number can predict distant recurrence in breast cancer patients^3^,^4^,^5^,^39^,^40^. The molecular characterization of the Notch1 - dependent Mena^INV^ expression shown here opens the possibility of developing additional markers that might be used in combination with TMEM sites to better predict the risk of distant metastatic recurrence of breast cancer patients and their response to treatment. We think that combining measures of Mena^INV^ expression with the presence of TMEM sites could be useful in determining the relative activity of TMEM sites in transendothelial migration and the response to inhibitors designed to suppress either Mena^INV^ expression and/or TMEM activity. Further work will be required to explore this possibility in patient cohorts of known outcome.

The spatial heterogeneity of expression of Mena^INV^ in primary mammary tumor cells and its consequences has been well described in previous studies^8^,^10^,^34^,^55^. However, the mechanisms regulating Mena^INV^ have remained unknown until now. Our finding of the increased expression of Mena^INV^ in response to Notch1 signaling between tumor cells and macrophages, but not between tumor cells and endothelial cells, restricts the origin of Mena^INV^ expression in the TMEM tumor cell, and therefore invadopodium assembly^34^, to the macrophage-tumor cell interaction. This leaves open the question about the myeloid cell type specificity of the induction of Mena^INV^ expression and invadopodia in tumor cells. In this regard neutrophils have been tested for their ability to induce invadopodia and transendothelial migration in a previous study and fail to do either^13^. Of relevance to the macrophage subtypes involved in tumor cell invasion and intravasation, the myeloid cells associated with tumor cells in mammary tumors during invasive migration resulting in intravasation^56^ are CD11b positive (a classical myeloid lineage marker) and GR1 negative (neutrophil marker) further excluding neutrophils from association with these particular tumor cells^57^. Furthermore, the CD11b positive tumor cell associated myeloid cells have been expression profiled to determine their identity as invasive macrophages^57^. High resolution intravital imaging has shown that transendothelial migration of tumor cells resulting in intravasation in mammary tumors occurs only in association with these invasive macrophages at TMEM. Furthermore, conditional depletion of the macrophages and/or knock out of the macrophage specific VEGF gene completely blocks intravasation *in vivo*^2^ further implicating macrophages in intravasation.

There is significant interest in targeting Notch1 signaling for the treatment of a number of cancers but previous studies have shown chronic Notch inhibition can lead to detrimental secondary effects and in some cases increased vascular tumor development^21^,^58^. Therefore, further study of the mechanisms of Notch1 signaling in breast cancer progression, as we show here, might lead to the identification of novel therapeutic targets within the Notch1 signaling cascade that might be better tolerated in patients.

## Methods

### Cell Lines and Reagents

MDA-MB-231 cells were cultured in DMEM supplemented with 10% FBS and antibiotics. MDA-MB-231 cells were serum-starved in DMEM supplemented with 0.5% FBS/0.8% BSA 16 h before macrophage induction studies. MTLn3 cells, derived from the 13762NF rat mammary adenocarcinoma, were cultured in α-MEM supplemented with 5% FBS and antibiotics. MTLn3 cells were serum-starved in α-MEM supplemented with 0.5% FBS/0.8% BSA 4 h before macrophage induction studies. BAC1.2F5 cells were cultured in MEM supplemented with 10% FBS, 2 mm l-glutamine, 22 μg/ml l-asparagine and 3000 U/ml of purified human recombinant CSF-1 (generously provided by Richard Stanley, Albert Einstein College of Medicine). Human umbilical vein endothelial cells (HUVECs, Lonza, Allendale, NJ, USA) were cultured in EGM-2 (Lonza, Allendale, NJ, USA) and only used between passage 1–4. DAPT (Sigma 10 μM) was used for gamma-secretase inhibition experiments as indicated in the results. When used, the DAPT or vehicle was added at the beginning of the experiments. Notch1 function blocking antibody (R&D Systems) was used *in vitro* at 5 μg/mL and *in vivo* via intraperitoneal injection at 1 mg/kg.

### siRNA

Control non-silencing siRNA was from Qiagen. Human-specific Notch1 siRNA pool was from Dharmacon and panMena and Mena^INV^ siRNA from Ambion^1^. A total of 1 × 106 MDA-MB-231 cells were transfected with 2 μm siRNA using the Lonza Nucleofection Kit V 72 h before each experiment. Immunoblot analysis and/or qPCR were performed to confirm knockdown for each experiment.

### Assay for Detection of Invadopodia

The 405 gelatin-labeled Mattek dishes were prepared as previously described^43^,^59^. Tumor cells were plated in complete media for 6 h on the Alexa 405-labeled gelatin dishes. Dishes were fixed and immunostained for cortactin and Tks5 as previously described. Cells were imaged on a wide-field microscope (Inverted Olympus IX70) and images were acquired with a cooled CCD camera (Sensicam QE cooled CCD camera) with a 60 × NA = 1.4 oil immersion objective using IP Laboratory 4.0 software. Invadopodia were detected as punctate structures that were positive for both cortactin and tks5 and capable of degrading Alexa 405-gelatin.

To detect macrophage-induced invadopodia, MDA-MB-231 cells were serum-starved for 16 h. BAC1.2F5 cells were cell tracker-labeled (CMPTX, Invitrogen). A total of 25 K MDA-MB-231 cells were incubated with 125 K BAC1.2F5 cells in serum-starvation media on 405-labeled gelatin-coated dishes for 6 h, fixed and immunostained for invadopodium markers as described above. For MTLn3 experiments, tumor cells were serum starved for 4 hrs before being plated with BAC1.2F5 cells for 6 hrs as described above.

### qPCR

qRT-PCR for Mena splice variants was performed as described previously^35^. Briefly, the data analysis was conducted using the ΔΔCt method in which all *MENA* Ct values in the carcinoma samples were first normalized to *GAPDH*. qRT-PCR analyses were performed using a SyBR Green kit (Qiagen) and analyzed with a Qiagene Rotor Gene-Q detector and associated software.

### Fine Needle Aspiration Biopsy

For primary cancer cells from two human invasive ductal carcinomas fine needle aspiration (FNA) was completed as previously described^1^. Briefly, lumpectomy and mastectomy specimens received at the Albert Einstein College of Medicine/Montefiore Medical Center, Moses and Weiler Divisions for pathological examination were used for FNA-based tissue collection under institutional review board approval. Four to five FNA aspiration biopsies per tumor were performed on grossly visible lesions using 25-gauge needles.

The TN human tissue transplant HT17 was previously described^1^,^37^. Briefly, the tumor originated from human patient samples and have since only been propagated in SCID mice. Tumors were harvested once they reached 1- to 1.2-cm diameter. Cells were obtained by FNA from human tumors grown in mice. All procedures were conducted in accordance with the NIH regulations and approved by the Albert Einstein College of Medicine animal use committee.

### FISH

#### *In Situ* Probes

48 oligodeoxynucleotide probes for MENA were designed with online Stellaris RNA FISH probe designer (Biosearch Tehchnologies). Each probe was 20 nt long and contained a 5′ amino-modified nucleotide that was chemically coupled to CAL Fluor 610 fluorescent dye. The sequences for probes used to detect MENA mRNA are provided in Supplementary Information.

#### *In Situ* Hybridization

For FISH experiments, MDA-MB-231 cells we starved overnight as described above. Tumor cells were plated in the presence or absence of BAC1.2F5 macrophages that were labeled with cell-tracker red on glass coverslips for 1 h then fixed with 4% paraformaldehyde for 20 minutes at room temperature. After washing away the fixative, the cells were stored in 70% (v/v) ethanol at 4 °C. Prior to hybridiazation, stored coverslips were washed with 1 × PBS and pretreated with 10% formamide/2 × SSC at room temperature for 5 minutes. The converslips were then inverted onto 20 μl of hybridization solution containing MENA FISH probes (0.125 μM), dextran sulfate (10%), 2xSSC, 10% formamide, *E. Coli* tRNA (1 mg/ml), vanadyl ribonucleoside complex (0.2 mg/ml), and bovine serum albumin (0.2 mg/ml). The cells were hybridized for 3 hours at 37 °C and washed with 10% formamide/2xSSC. The nuclei were stained with DAPI and the coverslips were then mounted with ProLong Gold antifade reagent (Invitrogen).

#### Image Acquisition and Analysis

Images were acquired on an Olympus BX61 epi-fluorescence microscope with an UPlanApo 60x, 1.35 numerical aperture oil immersion objective (Olympus). X-Cite 120 PC (EXFO) light source was used for illumination with filter sets 31000 (DAPI), 41001 (Cell-tracker Green), and SP-103v1 (CAL Fluor 610) (Chroma Technology). Vertical stacks of 30 images with a Z step size of 0.2 μm were acquired using a CoolSNAP HQ camera (Photometrics) with 6.4 μm pixel size CCD. IPLab (BD Biosciences) software platform was used for instrument control as well as image acquisition. Automated detection and counting of mRNAs was performed by fitting Gaussians to fluorescent spots with FISH-quant as described previously^60^,^61^.

### Production of Mena^INV^ antibody

Chicken poly-clonal antibodies were generated by Covance. Animals were immunized with a peptide containing the unique Mena^INV^ INV exon sequence.

Western blots of cell lysates from MDA-MB-231 cells expressing either GFP or GFP- Mena^INV^, when stained with the affinity purified anti-Mena^INV^ IgY, contain a single faint endogenous Mena^INV^ band in both cell types as described previously^55^ and a more intense band for GFP- Mena^INV^ in the over-expressing cells consistent with the specificity of this antibody (Supplemental Figure 4A). In addition, *in situ* immunofluorescent staining of mammary tumor tissue from WT and Mena Null PyMT mice demonstrate specificity of the antibody, where the WT tissue shows the presence of Mena^INV^ positive tumor cells while there is no staining above background in the Mena Null tissue^62^ (Supplemental Figure 4B).

### Transendothelial Migration Assay (iTEM)

The transendothelial migration assay was performed as described previously^1^,^13^ and briefly described here. The transwell was prepared so that tumor cell transendothelial migration was in the intravasation direction (from subluminal side to luminal side of the endothelium). We measure transendothelial migration as the intravasation-directed transendothelial migration (iTEM) from the tissue to the blood side of the endothelium. To prepare the endothelial monolayer, the underside of each transwell was coated with 50 μl of Matrigel (2.5 μg/ml; Invitrogen). Approximately 100,000 HUVEC cells were plated on the Matrigel coated underside of the transwells. Transwells were then flipped into a 24-well plate containing 200 μl of EGM-2 and monolayers were formed over a 48 hours period. The integrity of the endothelium used in this assay has been validated using electrical resistance and blockade of diffusion of small molecules^13^. Macrophages and tumor cells were labeled with cell tracker dyes (CMFDA, CMPTX from Invitrogen) before the experiment. Then, 15,000 macrophages and 37,500 tumor cells were added to the upper chamber in 200 μl of DMEM supplemented with 0.5% FBS while the bottom chamber contained EGM-2 supplemented with 3000 u/ml of CSF-1. After 18 hours of transmigration, the tranwells were fixed and stained for ZO-1 as previously described. Transwells were imaged using a Leica SP5 confocal microscope using a 60 × 1.4NA objective and processed using Image J [National Institutes of Health (NIH)]. Quantitation was performed by counting the number of tumor cells that had crossed the endothelium within the same field of view (60x) and represented as normalized values from at least 3 independent experiments. The quantitation of this assay is across at least 3 independent experiments, with 12 fields counted per transwell and transwells done in duplicate for each experiment.

### *In vivo* circulating tumor cells and photo-converted tumor cell dissemination assay

All procedures involving animals were conducted in accordance with NIH regulations, and approved by the Albert Einstein College of Medicine Animal Use Committee.

MTLn3 cells (parental and Dendra2 expressing) were injected into the mammary glands of 5 to 7 well old SCID mice and allowed to grow tumors for 3 weeks, until the tumors reached approximately 0.8–1.0 cm. Circulating tumor cell count was determined as previously described^36^. Briefly, 1 ml of blood was drawn from the right ventricle of anesthetized mice and plated in α-MEM media supplemented with 20% FBS. Tumor cell were counted as plated cells.

For the *in vivo* dissemination assay, a mammary imaging window was implanted and dissemination was measured as previously described^35^,^36^,^38^. Briefly, two days after the implantation of the imaging window regions of the tumor located adjacent to blood vessels were photo-converted. Approximately 5 regions were converted per tumor. After the photo-conversion and imaging of the regions (time 0), mice were treated with 1 mg/kg control IgG or Notch1 function blocking IgG (R&D Systems). The photo-converted regions were imaged at 24 hr, 48 hr, and 72 hr post treatment to determine the number of tumor cells that remained in the photo-converted primary tumor. The number of photo-converted cells remaining were counted at each time point and represented as the percent of photo-converted cells remaining compared to time 0. It should be noted that we have previously shown in the mouse mammary tumor model used in this study that the disappearance of photo-converted cells from the primary tumor gives rise to a disseminating population of tumor cells that is observed to arrive in distant organ sites seeding new metastases^14^. Furthermore, the tumor cells that disseminate from the photo-converted site are always associated with blood vessels^38^ and require functional invadopodia to disseminate^14^. In addition, the imaging method used to document the disappearance of photo-converted cells detects and counts tumor cells that simply “disperse” away from the conversion site ensuring that the disappearance of converted cells requires their dissemination from the primary tumor.

## Additional Information

**How to cite this article**: Pignatelli, J. *et al*. Macrophage-dependent tumor cell transendothelial migration is mediated by Notch1/Mena^INV^-initiated invadopodium formation. *Sci. Rep.* **6**, 37874; doi: 10.1038/srep37874 (2016).

**Publisher's note:** Springer Nature remains neutral with regard to jurisdictional claims in published maps and institutional affiliations.

## Supplementary Material

Supplemental Figures
