# Supplementary material for: Macrophage-dependent tumor cell transendothelial migration is mediated by Notch1/MenaINV-initiated invadopodium formation
Source: Sci Rep. 2016 Nov 30;6:37874. doi: 10.1038/srep37874 (PMC5129016; doi:10.1038/srep37874)
Supplement: Supplemental Figures [file srep37874-s1.doc]

**Macrophage-dependent tumor cell transendothelial migration is mediated by Notch1/Mena^INV^ -initiated invadopodium formation**

Jeanine Pignatelli^1,2,4^#*, Jose Javier Bravo-Cordero^1,2,5^#, Minna Roh-Johnson^1,2,6^, Saumil J. Gandhi^1^, Yarong Wang^1,2^, Xiaoming Chen^1^, Robert J. Eddy^1^, Alice Xue^1^, Robert H. Singer^1^, Louis Hodgson^1,2^, Maja H. Oktay^1,3^*, and John S. Condeelis^1,2^*

^1^Department of Anatomy and Structural Biology Albert Einstein College of Medicine of Yeshiva University, Bronx, NY 10461, ^2^ Gruss Lipper Biophotonics Center Albert Einstein College of Medicine of Yeshiva University, Bronx, NY 10461, ^3^Department of Pathology Albert Einstein College of Medicine of Yeshiva University, Bronx, NY 10461, ^4^Current Address: Pfizer Oncology Targeted Therapeutics Division, 401 N Middletown Rd, Pearl River, NY 10965, ^5^Current Address: Division of Hematology and Oncology, Department of Medicine, Mount Sinai School of Medicine, Tisch Cancer Institute, New York, NY, ^6^Current Address: Fred Hutchinson Cancer Research Center, Basic Sciences Division, 1100 Fairview Ave N, Seattle, WA 98109

# These authors contributed equally.

*Corresponding authors

*Correspondence to jeanine.pignatelli@pfizer.com, maja.oktay@einstein.yu.edu and john.condeelis@einstein.yu.edu


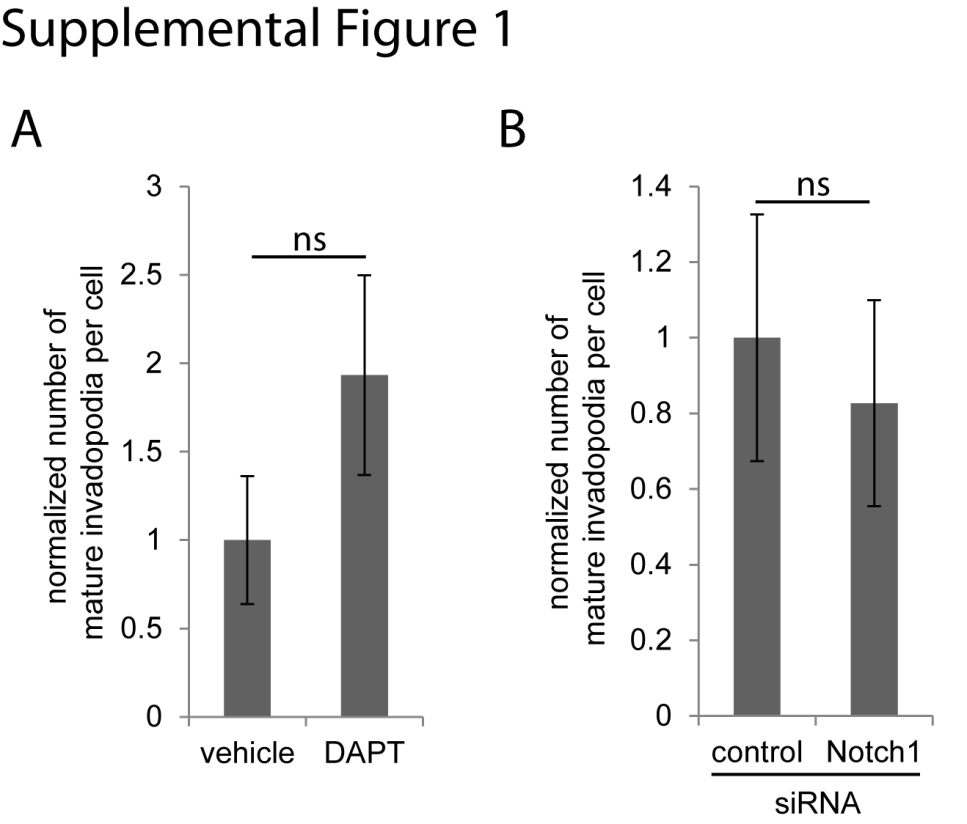


**Supplemental Figure 1: Notch1 is not required for the formation of spontaneous tumor cell invadopodia in the absence of macrophages. (A)** Quantitation of the number of mature invadopodia per cell in MDA-MB-231 cells plated on gelatin with the addition of vehicle or DAPT γ- secretase inhibitor. **(B)** Quantitation of the number of mature invadopodia per cell in MDA-MB-231 cells on gelatin treated with control or Notch1 siRNA. *ns = not significant*.

D

**
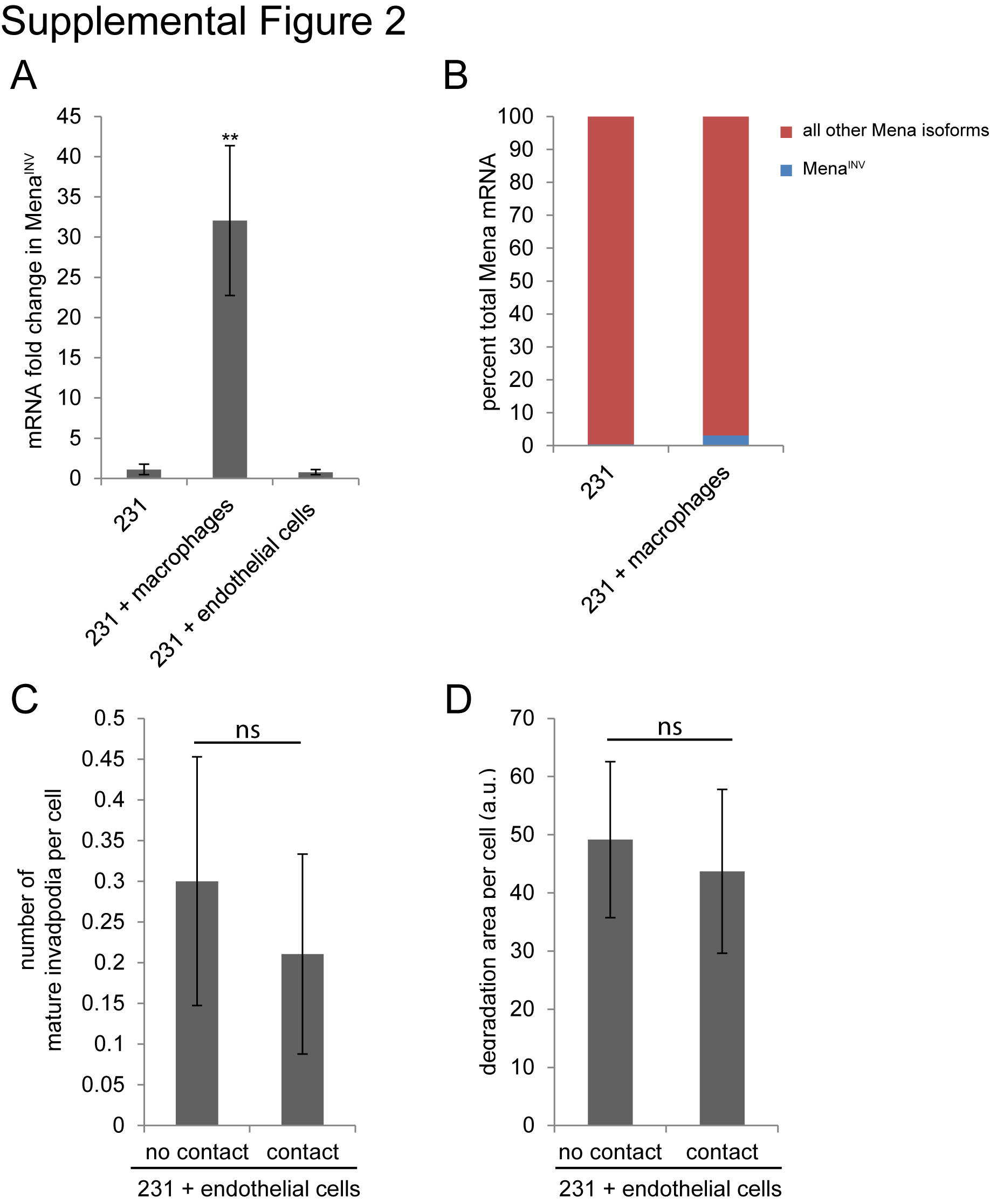
**

**Supplemental Figure 2: Macrophages are the specific TMEM cell that induces Mena^INV^ expression in tumor cells. (A)** Mena^INV^ expression occurs only in response to tumor cell contact with macrophages. mRNA fold expression change is shown in response to co-culture of tumor cells with either tumor cell (231), macrophages or endothelial cells. **(B)** Quantification of the percent of Mena^INV^ isoform mRNA compared to the total of all Mena isoforms (panMena) in MDA-MB-231 cells plated alone or with BAC1.2F5 macrophages indicating that Mena^INV^ is present as less than 5% of panMena. **(C)** Quantitation of the number of mature invadopodia per cell in MDA-MB-231 tumor cells plated with HUVEC primary endothelial cells with the two cell types either not in contact or in contact with each other. **(D)** Quantitation of the area of matrix degradation in MDA-MB-231 cells plated with HUVEC primary endothelial cells with the two cell types either not in contact or in contact with each other. *ns = not significant*. ***P* < 0.005


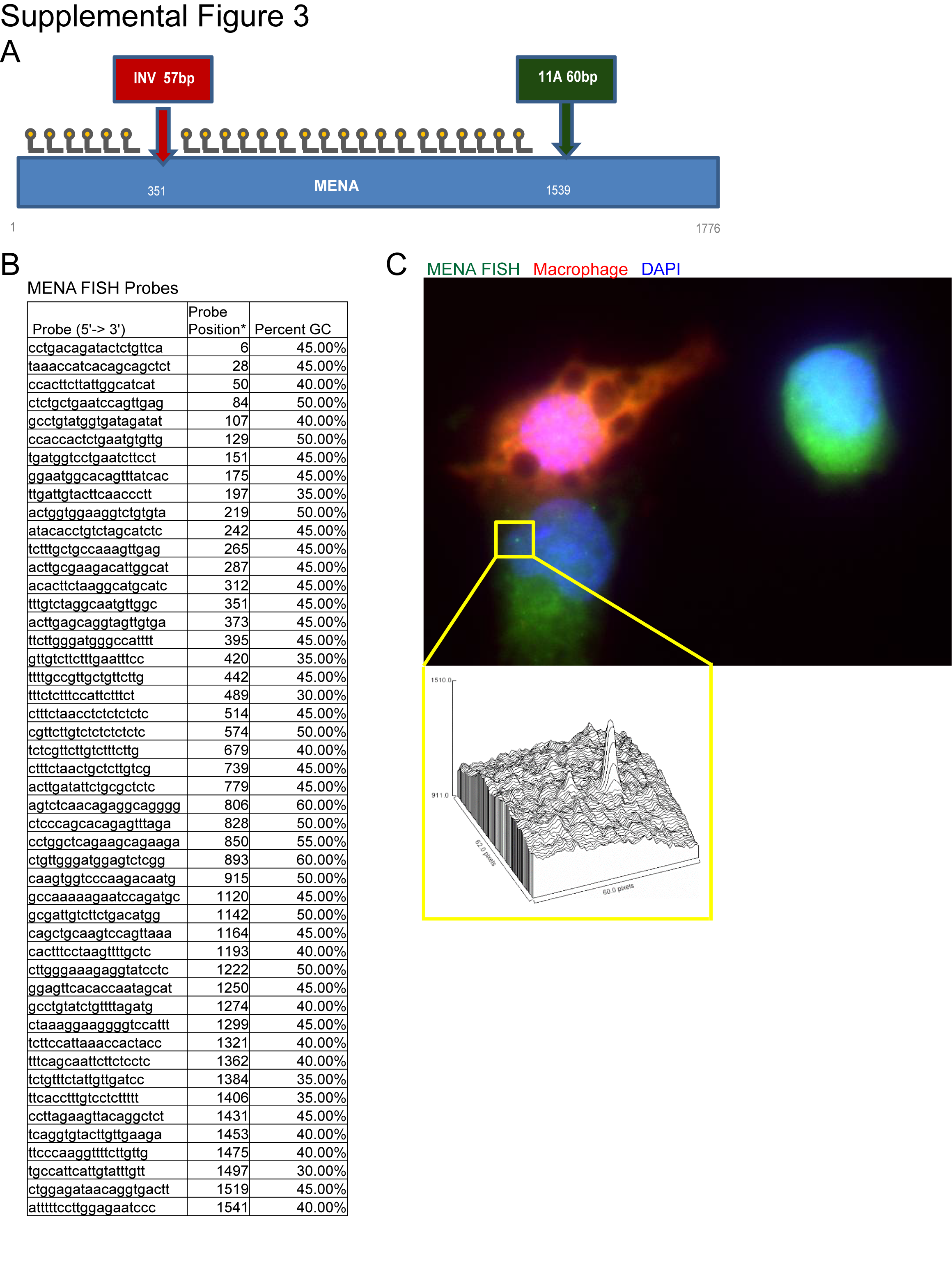


**Supplemental Figure 3: Design of MENA FISH probes.** (A) Schematic of FISH probes binding on MENA RNA. (B) Table of MENA FISH probes used (probe sequence, position on MENA, and percent GC). (C) MENA FISH in MDA-MB-231 cells with and without contact with macrophages (green is MENA FISH probes, macrophages are labeled with cell tracker red and blue is DAPI). Yellow box demonstrates active transcription site with florescence intensity above background.


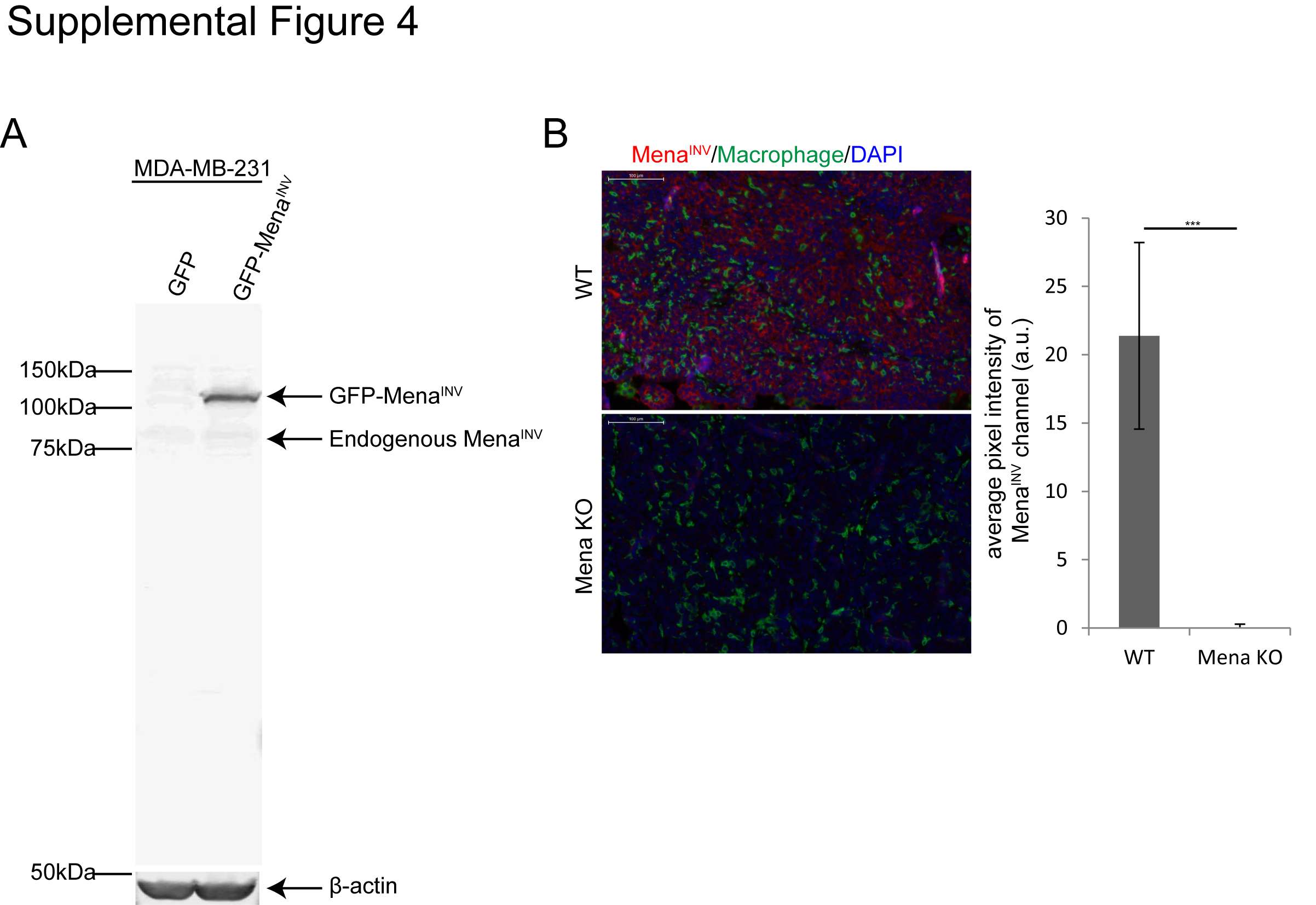


**Supplemental Figure 4:** **Design and validation of Mena^INV^ antibody. (A)** Western blot with Mena^INV^ specific antibody and β-actin of lysates from MDA-MB-231 GFP and GFP-Mena^INV^ expressing cells. **(B)** Tissue immunofluorescence (red) of sections from PyMT tumors of WT and Mena-null (MENA knock out) mice showing the presence and absence of staining, respectively. Graph quantifies the average pixel intensity of Mena^INV^ staining in PyMT tissues (background subtracted).

**P<* 0.05, ***P* < 0.005, ****P* < 0.0005
